# Tele-Coaching Korean Parents for Improving Occupational Performance of Toddlers: Three Case Reports

**DOI:** 10.3390/children10030492

**Published:** 2023-03-02

**Authors:** Dabin Choi, Aeri Yu, Misun Kim, Eun Young Kim

**Affiliations:** 1 Department of ICT Convergence, Soonchunhyang University, Asan-si 31538, Republic of Korea; 2 Center of Sensory Integration toward Social and Occupational Being, Seoul 04061, Republic of Korea; 3 Department of Occupational Therapy, Soonchunhyang University, Asan-si 31538, Republic of Korea

**Keywords:** tele-coaching, telehealth, Occupational Performance Coaching, parenting

## Abstract

Telehealth has been applied to occupational therapy practice since the COVID-19 pandemic, but no research has been conducted on the use of telehealth to improve the occupational performances of Korean children and parents. This study explored the possibility of tele-coaching parents to improve toddlers’ occupational performance and parenting competence in Korea. Three mothers of toddlers received Occupational Performance Coaching (OPC) via videoconference. The Canadian Occupational Performance Measure (COPM) and the Parenting Sense of Competence Scale (PSOC) were used pre- and post-intervention to measure the occupational performances of the toddlers and parents and parenting competence. Post-intervention interviews were conducted to explore the parents’ experiences with the tele-coaching and analyzed by content analysis. Most of the COPM scores showed a significant increase. The PSOC scores also increased. The mothers reported their learning, the changes in their children, the appropriateness of the coaching, and the usefulness of the tele-coaching delivery. The findings demonstrate the potential of tele-coaching as a practical intervention for Korean children and parents.

## 1. Introduction

Coaching is a client-centered approach in occupational therapy to help clients achieve occupational goals [1]. Coaching has been used to improve the occupations of children with disabilities [2] and adults with health conditions [3,4]. In particular, interventions through parent coaching can enhance parental insight and learning [5,6] and children’s development [7], social interactions [8], occupational performance [9], and participation in daily activities [10].

Occupational Performance Coaching (OPC) [11] has been applied to parent coaching [12,13]. It is an occupation-centered intervention to facilitate children’s occupational performance by addressing parents’ learning needs. OPC is based on a family-centered approach that respects parents’ knowledge and decisions. By working with parents, OPC aims to enable children and parents to engage optimally in their occupations.

OPC intervention can improve children’s occupations (e.g., playing, social interactions, riding a bike, and handwriting) and parenting performance (e.g., interactions with their children and supporting their children’s learning) [14]. Through OPC, parents reported that they learned better skills and strategies, gained insights into themselves and their children, and became more confident and calm [6]. OPC can be effective for children with occupational performance challenges, cerebral palsy, intellectual disabilities, autism spectrum disorder, developmental delay, and dyslexia [9,14,15,16,17,18,19,20]. Improvements by OPC were observed not only for the goals targeted in an intervention but also for goals that were not addressed, indicating a generalized effect [9].

Parent coaching can be delivered by various methods, such as in-person interventions [21], in-person services supplemented with phone sessions [5,14], and telehealth using videoconferencing [19,20,22]. A recent study explored the feasibility of OPC in Hong Kong during the COVID-19 pandemic, where the intervention was delivered face-to-face or via the internet [16]. The parents in that study mentioned the advantages of online meetings, which included saving on travel time and showing the home environment. The effectiveness of OPC via videoconference for families of children with autism was comparable to that of face-to-face OPC [19]. Telehealth has become more emphasized in the era of the COVID-19 pandemic [23,24]. To meet the demands of the pandemic, this study provided OPC through tele-coaching.

To our knowledge, no studies have been reported on OPC or tele-coaching for the occupational performance of Korean children and parents. Other coaching methods in occupational therapy have recently been explored for Korean children with disabilities [21,25], suggesting the potential of occupation-centered coaching for facilitating children’s occupations in Korea. These previous studies raised the possibility that OPC, which has not yet been applied in Korea, may enhance the occupational performance of Korean children and parents. Recent studies showed that OPC via videoconference was feasible [19,20], which implies the applicability of OPC-based tele-coaching in Korea. The primary aim of this study was to investigate whether tele-coaching based on OPC could improve the occupational performance of Korean toddlers and parents.

Previous studies revealed the efficacy of OPC for children aged three years and older [9,14,15,16,17,18,19,20]. However, no studies have applied OPC to children younger than three years of age. Parent coaching has been a useful intervention for young children [8,22], suggesting that OPC could be applied to toddlers. In early childhood, the parent’s role is important in supporting the participation of young children, who develop rapidly [26]. Parent coaching in early intervention has been used by occupational therapy practitioners [27]. Therefore, toddlers were included in this study to examine whether young children can benefit from OPC.

To examine the applicability of OPC-based tele-coaching, we measured the occupational performance of the toddlers and parents and parenting competence. We also explored the parents’ experiences with tele-coaching. The findings from this study contribute to the use of tele-coaching for occupational performance improvement in Korea by demonstrating the first evidence that OPC-based tele-coaching may be applicable to Korean toddlers and parents.

## 2. Methods

### 2.1. Study Design

This study used a descriptive case study method [14,28]. The study was approved by the Institutional Review Board of Soonchunhyang University. The research was conducted in a community setting from October 2021 to December 2021.

### 2.2. Participants

Three mother–toddler pairs were included in this study. They were recruited from a waiting list at the community developmental center, which provides intervention services for occupational performance and sensory integration. The inclusion criteria were (1) toddlers on the waiting list of the community developmental center, (2) those who were younger than three years old, and (3) parents who completed four pre-planned coaching sessions. Written informed consent was obtained from all parents.

### 2.3. Procedure

**Figure 1:**
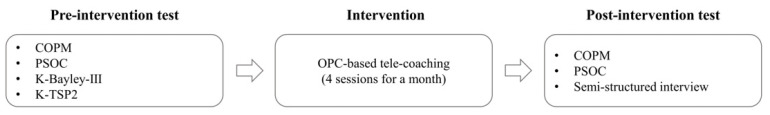
The intervention procedure. COPM: Canadian Occupational Performance Measure; PSOC: Parenting Sense of Competence Scale; K-Bayley-III: Korean Bayley Scales of Infant and Toddler Development, Third Edition; K-TSP2: Korean Toddler Sensory Profile 2; OPC: Occupational Performance Coaching.

The interventional procedure consisted of one pre-intervention test, four tele-coaching sessions, and one post-intervention test (Figure 1). All coaching and interview sessions were conducted individually via the Zoom online platform.

During the pre-intervention test, the first author asked the mothers to identify occupational goals using the Canadian Occupational Performance Measure (COPM) [29]. The mothers completed the Parenting Sense of Competence Scale (PSOC) [30,31,32]. They also rated their children’s function and behaviors using the social–emotional and adaptive-behavior scales of the Korean Bayley Scales of Infant and Toddler Development, Third Edition (K-Bayley-III) [33,34] and the Korean Toddler Sensory Profile 2 (K-TSP2) [35,36], which provided information about development and the sensory-processing patterns of the children. The questionnaires, including the PSOC, the K-Bayley-III, and the K-TSP2, were mailed to the parents and the completed forms were received before coaching. The pre-intervention test was followed by four tele-coaching sessions of one hour each about once a week for one month. The coach, the second author, with fifteen years of experience as an occupational-therapy practitioner, was different from the tester. The COPM and the PSOC were re-administered in the post-intervention test to examine the improvements from tele-coaching. During the post-intervention, the mothers’ experiences with OPC-based tele-coaching were also explored through a semi-structured interview.

### 2.4. Intervention Protocol

The intervention was based on OPC [12,13]. OPC is a family-centered, occupation-centered, and problem-solving practice to improve the occupational performances of children and their parents. It has three enabling domains: emotional support, information exchange, and a structured process. The emotional support domain includes listening, empathizing, reframing, guiding, and encouraging. A coach provides emotional support to help parents to become ready and to persist in solving problems by viewing challenges in constructive ways. In the information exchange domain, the coach and parents provide knowledge together to identify and solve problems. In collaborative performance analysis, the coach determines the child’s current and desired performances, the bridges and barriers between them, and the parent’s needs. The exchanged information can include typical development, health conditions and impairments, teaching and learning strategies, specialized strategies, and community resources and entitlements. The last domain is a structured process aligned with a problem-solving approach. The steps of the structured process are setting goals, exploring options, planning action, carrying out plans, checking performance, and generalizing.

### 2.5. Measurements

#### 2.5.1. Canadian Occupational Performance Measure

The COPM is used to identify clients’ perceptions of their occupations [29]. The client reports the occupational performance problems and their importance. Next, the client rates the performance and satisfaction of the five most highly prioritized activities using a 10-point Likert scale. A change of 2 or more points is considered clinically significant progression. The COPM was used to identify the occupations that needed to be addressed and to measure changes according to tele-coaching.

#### 2.5.2. Parenting Sense of Competence

The PSOC assesses parenting satisfaction and efficacy by self-reporting [30,31]. This study used 15 items of the Korean version of the PSOC [32] rated on a 5-point Likert scale. A high score on the PSOC indicates that a parent perceives competence in their parenting. The PSOC was applied to compare parenting competence before and after tele-coaching.

#### 2.5.3. Korean Bayley Scales of Infant and Toddler Development, Third Edition—Social–Emotional and Adaptive Behavior Scales

The K-Bayley-III assesses the developmental function of young children aged 16 days to 42 months and 15 days [33,34]. This study used the social–emotional and adaptive behavior scales to identify children’s socioemotional development and adaptive skills before tele-coaching. These scales are parent-reported questionnaires. The composite scores are categorized as “Extremely low” (69 or below), “Borderline” (70–79), “Low average” (80–89), “Average” (90–109), “High average” (110–119), “Superior” (120–129), and “Very superior” (130 and above).

#### 2.5.4. Korean Toddler Sensory Profile 2

The K-TSP2 assesses sensory processing patterns in 7- to 35-month-old toddlers by parent reports [35,36]. The K-TSP2 provides quadrant scores and sensory and behavioral scores. These scores are categorized into “Much less than others”, “Less than others”, “Just like the majority of others”, “More than others”, and “Much more than others” based on the frequency of the behavior described in the items. The K-TSP2 was used to evaluate the children’s sensory-related behaviors in daily life before tele-coaching.

#### 2.5.5. Guiding Questions for the Post-Intervention Interview

A post-intervention interview was performed to explore parents’ experiences with OPC-based tele-coaching. Guiding questions for the interview were constructed according to previous research that investigated the effectiveness of OPC [6,16]. A list of the questions is shown in Table 1.

### 2.6. Data Analysis

Descriptive analysis was performed on the quantitative measurements. Because this study included three cases, raw data of the COPM and the PSOC were described for each case. We calculated the average COPM scores for each participant and then the mean of the average scores across the participants. The PSOC scores were also averaged into the mean.

For the qualitative data, the post-intervention interview was analyzed by content analysis [37,38]. The interview transcripts were coded independently by two coders. The coders read the transcripts and inductively created codes. They then refined and condensed the codes by reading the transcripts again. The coders preliminarily generated categories and sub-categories. Next, the two coders met to determine the categories and sub-categories by reviewing the coded data. They discussed any discrepancies and developed a consensus on the categories and sub-categories. Using these consensus categories and sub-categories, the transcripts were coded independently again by two coders while considering alternative categories. The categories were finalized by consensus among the coders. We used Microsoft Excel to perform quantitative and qualitative analyses.

## 3. Results

### 3.1. Case Desciptions

Three pairs of mothers and children were included in this study (Table 2). The children were one girl and two boys aged 23 to 30 months. The mothers’ goals in the tele-coaching are shown in Table 3.

### 3.2. Comparison between Pre- and Post-Intervention Scores on the COPM and the PSOC

**Figure 2:**
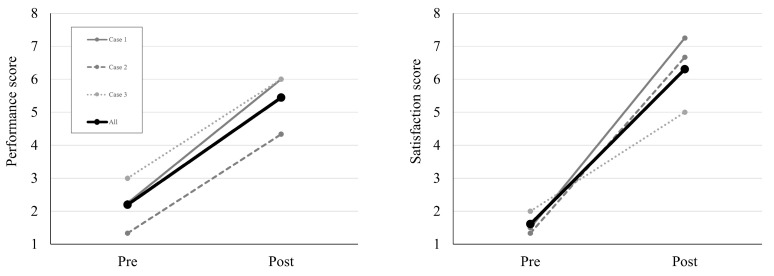
The change in the mean COPM-performance and satisfaction scores from pre- to post-intervention tests. COPM: Canadian Occupational Performance Measure.

Most of the COPM performance scores showed a change of two and more points, except for the scores for one goal (Table 3). Figure 2 shows the changes in the mean COPM scores. The mean performance score increased from 2.19 (0.83) to 5.44 (0.96). All the satisfaction scores of the COPM were clinically significantly changed. The mean satisfaction score increased from 1.61 (0.35) to 6.31 (1.17). These results indicate improvements in the occupational performances of the children and parents. The PSOC scores increased from 39 to 45 in Case 1, from 43 to 50 in Case 2, and from 50 to 56 in Case 3. The mean PSOC score increased from 44 (5.57) to 50.33 (5.50). These increases indicate that the mothers felt more competent at parenting after the intervention.

### 3.3. Parents’ Experiences with Tele-Coaching

All the mothers rated the question asking whether the intervention met their expectations with the maximum score of “expectations were exceeded a lot”. They also expressed willingness to recommend tele-coaching to other parents.

Four categories were revealed through the content analysis of the interview: (1) parents’ learning, (2) children’s changes, (3) perceived coaching characteristics, and (4) evaluation of tele-coaching delivery.

#### 3.3.1. Parents’ Learning

All the mothers reported that they gained insights into their children or learned skills/strategies to improve their children’s occupational performance through tele-coaching. For example, the mother of Case 2 stated: “My mindset has been changed. A new way to help my child was introduced. The new strategy is not totally different from the previous strategy. I just didn’t know how to do it before. I got practical support that I couldn’t get from a book”. The parents also mentioned that they encountered challenges when implementing the strategies. For example, the mother of Case 2 stated: “I almost gave up in the middle. Nevertheless, I tried a different strategy. It worked”. All the mothers reported that they came to feel positive emotions, such as comfort and confidence. For example, the mother of Case 1 commented, “I have felt comfort since I came to know my child’s temperament and sensitivity”.

#### 3.3.2. Children’s Changes

All the mothers reported that their child’s occupational performance was improved, which could be attributed to the tele-coaching. For example, the mother of Case 1 stated: “I could see my child’s changes because I learned”. The mother of Case 3 commented: “Since I applied what I was coached, interactions improved, and my child looked at me more”. All the mothers commented on their children’s positive emotions. For example, the mother of Case 1 reported: “My child feels understood by me”. The mother of Case 2 noted: “I think my child has will and self-esteem”.

#### 3.3.3. Perceived Coaching Characteristics

The mothers commented on the appropriateness of the coaching, describing mutual communication, timely intervention for the toddlers, and their role in implementing the strategies they learned. For example, the mother of Case 3 commented: “It was more effective because I did what a therapist had intervened with my child before”. All the mothers were satisfied with their relationship with the coach, mentioning that the coach acknowledged, encouraged, and guided them. For example, the mother of Case 1 stated: “It was really helpful that the coach complemented what I was doing…it would have been difficult for me if she had told me I was wrong… I really appreciate her encouragement”.

#### 3.3.4. Evaluation of Tele-Coaching Delivery

All the mothers stated that the videoconference coaching was useful because it allowed them to share materials, save on travel time, see each other, and easily receive services during the COVID-19 pandemic. Although most of the comments referred to the positive aspects of the tele-coaching, the mother of Case 1 pointed out a limitation of the communication-based online intervention. All the mothers considered the session duration (one hour) and frequency (about once a week) appropriate, but they wanted more sessions. The mothers suggested that the sessions need to be scheduled at a time in which distractions are not present.

## 4. Discussion

This study demonstrated that tele-coaching based on OPC is applicable to improve the occupational performances of young children and parents in Korea. The parents were satisfied with the OPC-based tele-coaching, reporting that they learned how to support their children’s occupations. Furthermore, the parents felt more competent in their parenting occupations after the intervention. These findings are aligned with previous studies revealing the effectiveness of OPC [9,14,15,16,17,18,19,20] and tele-coaching [19,20,22]. The present study provides evidence that OPC delivered via tele-coaching can be applied to Korean toddlers and parents.

The parents’ experiences with OPC via tele-coaching in this study were consistent with the themes found in previous studies [6,14,16]. Research implementing OPC, including this study, showed that parents gained insights or learned strategies/skills, and that this learning led to improvements in the occupational performances of children and parents. These studies also found that parents and children felt more positive emotions due to coaching. During the intervention, the parents experienced collaborative relationships with their coach and took an active role in implementing the strategies they learned.

A recent study in Korea reported the effectiveness of parent coaching that targeted the occupations of children with autism spectrum disorder [21]. The coaching consisted of family-centered management [39] and contextual intervention [5] based on sensory integration therapy. There was a difference in the delivery of the services between the previous study and the present study. The coach in the previous study visited the children’s homes, whereas the coach in the present study met with the parents online. This study was the first to focus on the occupation-centered tele-coaching of Korean parents.

This study utilized videoconferencing to deliver interventions to parents with young children. Recent studies found that videoconference-delivered OPC can improve the occupational performance of children with autism [19,20]. In a study conducted during the COVID-19 outbreak, two of four child–parent pairs participated in OPC sessions through Zoom, and the parent reported learning and changes in their children’s occupations [16]. Both previous studies and the current study demonstrated that videoconferencing was positively evaluated by parents in terms of saving travel time and sharing information, suggesting the applicability of OPC delivered via tele-coaching.

To determine whether the results of this study were comparable to those of previous OPC-based tele-coaching research, we reviewed the changes in COPM scores, the common measurement across studies. In the present study on Korean toddlers with developmental problems, the mean COPM performance and satisfaction scores increased by about three and four points, respectively. Similarly, the mean COPM scores increased by about three points in the Australian families of children with autism [19]. In a study conducted in Hong Kong, children with autism and developmental delays also showed changes of about two points [16]. Another recent study involving Iranian children with autism reported an increase in COPM scores of about two points [20]. Taken together, our study is consistent with previous studies showing that OPC-based tele-coaching could be useful in improving the occupational performance of children with developmental difficulties.

The participants in this study were the parents of toddlers aged 23 to 30 months, whereas previous studies using OPC included the parents of children aged three years and older [6,9,14,16,17,18,19,20]. The present study showed that OPC could be effective in toddlers by focusing on the occupational role of parents in supporting children’s participation and development. The parents in this study came to understand their young children and discovered solutions to challenging situations with this insight. As a result, parenting competence was increased. This finding suggests that OPC may be applicable to populations with challenges in their parenting occupations.

The present study can practically contribute to improving the occupations of Korean children and parents. First, this study demonstrated that OPC can be applied in Korea. The effectiveness of OPC for children and parents has been reported in various countries such as Australia [9,19], Canada [18], Hong Kong [16], India [17], and Iran [20]. Our study and previous studies suggest that OPC could be effective across cultures. Second, tele-coaching can be a feasible delivery method for OPC, especially during a pandemic. Telehealth has been emphasized in Korea, where the COVID-19 pandemic started early and daily life was substantially restricted [40]. Tele-coaching based on OPC in Korea can be comparable to that in Australia [19], Hong Kong [16], and Iran [20]. Third, the present study expanded the target of OPC by including toddlers. OPC was previously applied to children over three years of age [6,9,14,16,17,18,19,20]. The present study can be evidence to broaden the age range of OPC.

The present study also offers a theoretical contribution to support the self-determination theory underlying OPC [11,41,42]. The self-determination theory postulates that intrinsic motivation to engage in behaviors and activities increases when the three psychological needs of autonomy (feeling ownership of one’s goals and behaviors), relatedness (feeling connection with others), and competence (perceiving oneself as effective) are satisfied [43]. Our qualitative results revealed components of autonomy, relatedness, and competence. For example, the mothers reported experiences reflecting their sense of ownership of the strategies they applied (autonomy), the emotional support of the coach (relatedness), and problem solving (competence). These results provide additional evidence that OPC addresses the basic psychological needs conceptualized in the self-determination theory.

This study is a starting point for the use of tele-coaching to improve the occupational performances of parents and young children in Korea. However, this study has limitations that require further research. The study described only three cases. A larger number of participants should be included in future studies with a higher level of evidence, such as a randomized controlled trial. Further studies with larger samples may also examine which factors are related to the effectiveness of tele-coaching. Additionally, it is necessary to increase the number of coaching sessions in further research, as suggested by the mothers.

## 5. Conclusions

This study demonstrated the application of tele-coaching based on OPC for children’s occupations, as well as parental learning and competence. The OPC improved the occupations of the toddlers and mothers, which was shown by the changes in the mean COPM performance score from 2.19 to 5.44 and the mean COPM satisfaction score from 1.61 to 6.31. The mothers’ parenting competence was also enhanced, as the mean PSOC score changed from 44 to 50.33. In addition, all the mothers were satisfied with the tele-coaching and were willing to recommend it to other parents.

The results of this preliminary investigation suggest that tele-coaching may be applied to improve the occupational performances of parents and children in Korea. This study contributes to the evidence that OPC can be applicable across cultures and that tele-coaching is a feasible practice with which to deliver OPC. In addition, our findings on the toddlers showed that OPC can enhance the occupations of younger children. The improvement in the occupational performances of Korean toddlers and parents may be attributed to the effects of OPC on autonomy, relatedness, and competence, which supports the self-determination theory. This study is expected to be the basis for further research on the occupation-centered tele-coaching of Korean parents with young children.

## Figures and Tables

**Table 1:** Guiding questions for the post-intervention interview. Reprinted/adapted with permission form Refs. [6,16].

Questions
“What did you like most during the parent-coaching period?”
“What did you like least during the parent-coaching period?”
“What did you learn during the parent-coaching period?”
“What are the key things you learned from the coaching sessions?”
“Did you learn anything that surprised you or that you hadn’t really expected? (yes/no). If yes, what was this?”
“What did you already know about helping your child to learn that the sessions prompted you to focus on more?”
“During the parent-coaching period, what difficulties did you encounter?”
“How would you describe your relationship with the coach?”
“Did the intervention meet your expectations?” (1–5 Likert scale; 1 = ‘expectations not at all met’; 2 = ‘expectations somewhat met’; 3 = ‘expectations met to a great extent’; 4 = ‘expectations were exceeded a little’; 5 = ‘expectations were exceeded a lot’).
“How has the parent-coaching helped your child to engage in daily activities?”
“How has the parent-coaching helped to facilitate your child’s development?”
“How has the parent-coaching helped to improve your child’s psychosocial aspects?”
“How has the parent-coaching affected your psychosocial aspects?”
“What did you think about the coaching schedule?”
“What did you think of the number of sessions being four?”
“What did you think about the sessions being once a week?”
“What did you think about each session being one hour long?”
“What did you think about the online video meeting (Zoom) as the delivery method?”
“Would you recommend the parent-coaching to other parents in need? If yes, how would you explain the intervention to them? If no, why would you not recommend it?”
“What improvements to the parent-coaching would you suggest if it were to be applied in Korea in the future?”

**Table 2:** Participant characteristics.

	Case 1	Case 2	Case 3
Child’s age (months)	20	30	23
Child’s gender	Female	Male	Male
Diagnosis/problem	Language delay	Developmental disorder	Developmental delay
K-Bayley-III			
Social–emotional scale	Average	Borderline	Extremely Low
Adaptive behavior scale	Low average	Extremely low	Borderline
K-TSP2			
Seeking	Just like the majority of others	Much less than others	Just like the majority of others
Avoiding	More than others	Just like the majority of others	Just like the majority of others
Sensitivity	Just like the majority of others	Less than others	Just like the majority of others
Registration	Just like the majority of others	Just like the majority of others	More than others

K-Bayley-III: Korean Bayley Scales of Infant and Toddler Development, Third Edition; K-TSP2: Korean Toddler Sensory Profile 2.

**Table 3:** Goals and scores of the Canadian Occupational Performance Measure.

Case	Goal	Performance			Satisfaction		
		Pre	Post	Change	Pre	Post	Change
Case 1	The mother engages in the child’s play appropriately and strengthens attachment.	4	8	4	1	8	7
	The child establishes a sleep routine and goes to sleep early.	3	3	0	3	6	3
	The child stays calm and walks by herself when going out in the community.	1	9	8	1	9	8
	The child expresses herself appropriately without throwing objects.	1	4	3	1	6	5
Case 2	The child eats snacks independently using a fork and spoon.	1	4	3	1	6	5
	The mother plays with the child interactively.	2	4	2	2	6	4
	The child uses the toilet instead of diapers.	1	5	4	1	8	7
Case 3	The child plays in various ways.	4	6	2	2	5	3
	The child engages in social interaction with others.	2	6	4	2	5	3

## Data Availability

The data that support the findings of this study are available from the corresponding author upon reasonable request. The data are not publicly available because they concern the privacy of children.
